# Exploring Generative Pre-Trained Transformer-4-Vision for Nystagmus Classification: Development and Validation of a Pupil-Tracking Process

**DOI:** 10.2196/70070

**Published:** 2025-06-06

**Authors:** Masao Noda, Ryota Koshu, Reiko Tsunoda, Hirofumi Ogihara, Tomohiko Kamo, Makoto Ito, Hiroaki Fushiki

**Affiliations:** 1 Department of Otolaryngology, Mejiro University Ear Institute Clinic, 320 Ukiya, Iwatsuki-ku, Saitama-shi, Saitama, 339-8501, Japan, 81 48 797 3341; 2 Department of Otolaryngology, Jichi Medical University, Shimotsuke, Japan

**Keywords:** nystagmus, GPT-4Vision, generative AI, deep learning, dizziness, artificial intelligence

## Abstract

**Background:**

Conventional nystagmus classification methods often rely on subjective observation by specialists, which is time-consuming and variable among clinicians. Recently, deep learning techniques have been used to automate nystagmus classification using convolutional and recurrent neural networks. These networks can accurately classify nystagmus patterns using video data. However, associated challenges including the need for large datasets when creating models, limited applicability to address specific image conditions, and the complexity associated with using these models.

**Objective:**

This study aimed to evaluate a novel approach for nystagmus classification that used the Generative Pre-trained Transformer 4 Vision (GPT-4V) model, which is a state-of-the-art large-scale language model with powerful image recognition capabilities.

**Methods:**

We developed a pupil-tracking process using a nystagmus-recording video and verified the optimization model’s accuracy using GPT-4V classification and nystagmus recording. We tested whether the created optimization model could be evaluated in six categories of nystagmus: right horizontal, left horizontal, upward, downward, right torsional, and left torsional. The traced trajectory was input as two-dimensional coordinate data or an image, and multiple in-context learning methods were evaluated.

**Results:**

The developed model showed an overall classification accuracy of 37% when using pupil-traced images and a maximum accuracy of 24.6% when pupil coordinates were used as input. Regarding orientation, we achieved a maximum accuracy of 69% for the classification of horizontal nystagmus patterns but a lower accuracy for the vertical and torsional components.

**Conclusions:**

We demonstrated the potential of versatile vertigo management in a generative artificial intelligence model that improves the accuracy and efficiency of nystagmus classification. We also highlighted areas for further improvement, such as expanding the dataset size and enhancing input modalities, to improve classification performance across all nystagmus types. The GPT-4V model validated only for recognizing still images can be linked to video classification and proposed as a novel method.

## Introduction

Equilibrium function in vertigo practice can be evaluated through nystagmus assessment. Nystagmus is characterized by rhythmically repeated rapid and slow eye movements and serves as a valuable clinical indicator for diagnosing various neurological and vestibular disorders. Nystagmus can influence the normal function of the cerebellum, semicircular canals, and integrated eye movements, and thus is of great diagnostic and therapeutic importance [1-3]. The direction of nystagmus can be horizontal, vertical, or torsional (rotational), depending on the axis of the eye movement. The evaluation of nystagmus patterns provides essential insight into the function of the visual and vestibular systems by identifying underlying foci and guiding treatment strategies. Traditionally, nystagmus classification has relied heavily on subjective observation by trained specialists, which is time-consuming, prone to variability among clinicians, and can be difficult to perform in the emergency department [4]. Furthermore, advancement of diagnosis can contribute to improper treatment, increasing the risk of falls or decreasing daily physical activity levels [5,6].

In recent years, advances in artificial intelligence (AI) and machine learning technologies have provided promising means to capture eye movements [7,8] and automate nystagmus classification, thereby improving the accuracy and efficiency of diagnosis [9,10]. In particular, deep learning methods such as convolutional and recurrent neural networks are increasingly being used to analyze medical imaging data, including videos that capture eye movements [11,12]. Although these techniques have shown success in tasks such as image classification, object detection, and segmentation, applying deep learning to nystagmus classification remains challenging given the temporal changes inherent in eye movement patterns. Interestingly, recent studies have demonstrated promising outcomes by using deep learning techniques to annotate scenes or detect spatiotemporal features. This suggests that the potential of deep learning algorithms to classify nystagmus patterns based on their capture, and interpretation of these temporal characteristics requires sophisticated modeling methods that can effectively process ordinal data. Notably, several reports have used deep learning to enable a unified evaluation of other perceptions; however, creating models requires large amounts of data, and it is unclear how such models can be used.

A large-scale language processing model, known as a large language model (LLM) is a highly versatile system trained on extensive text data using a transformer architecture. It has demonstrated high accuracy in medical classification and text recognition [13]. In one such model, the Generative Pre-Trained Transformer (GPT), the advent of GPT-4Vision (GPT-4V) made it possible to combine image recognition by devising prompts and classification without requiring image training data [14,15]. Unlike convolutional neural networks or recurring neural networks, GPT-4V leverages in-context learning, allowing it to classify visual and multimodal data without extensive training datasets [16]. In this study, we developed a novel nystagmus classification approach that leverages the capabilities of the GPT-4V model. We aimed to develop a classification system that can accurately identify different nystagmus patterns and validate its accuracy by integrating GPT-4V with an eye movement tracking algorithm from eye movement video data. This study is one of the first to evaluate the feasibility of GPT-4V for nystagmus classification, particularly in scenarios where dataset limitations exist.

## Methods

**Figure 1.**
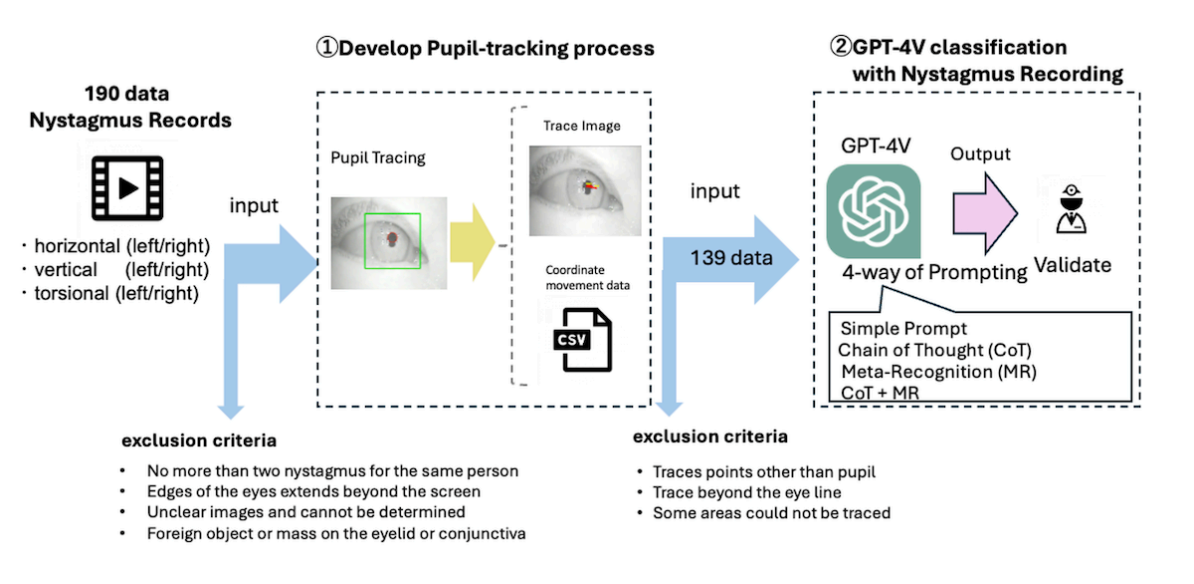
Study overview. GPT-4V: Generative Pre-trained Transformer 4 Vision.

In this study, we developed a pupil-tracking process using nystagmus recording videos and verified the accuracy of the optimization model using GPT-4V classification and nystagmus recording (Figure 1).

### Developing the Pupil Tracking Process

First, the eyeballs were recognized from the video data, and pupil movement co-ordinate data were created using an eye movement tracking algorithm that showed the eyeball trajectory. Video data were recorded using a charge-coupled device-based camera eyeball rotation imaging device, ET-60LW2 (Newopto), with a focal length of 6 mm, and a horizontal resolution >500 television lines. The sensor size is 1/3 inch and intermittent synchronous lighting occurred every 1/30 second. A single video contained at least three nystagmus in the same direction and was 3 to 5 seconds in duration.

Based on these data, 190 trace images were created for each video from patients whose main complaint was dizziness. Videos were created using the same algorithm used to generate trace images of the same individual, with no more than two nystagmus events. After applying the exclusion criteria, trace data were successfully generated for 139 patients. The exclusion criteria included cases in which the edges of the eyes extended beyond the screen, images that were too unclear to be evaluated, and the presence of foreign objects or masses on the eyelid or conjunctiva.

### Pupil Movement Tracking Algorithm

An eye movement tracking algorithm based on video data was developed using the Haar cascade classifier and OpenCV [17]. Although deep learning–based approaches may offer higher accuracy for detecting facial features, they often require extensive data or a pretrained model, as well as comparatively intensive inference processes. The outline of the pupil and its center within the screen were detected, enabling the tracking of the center’s coordinates. In contrast, the Haar cascade classifier, as part of the OpenCV suite, offers a more streamlined and efficient alternative, enabling the tracking of the eye’s trajectory at a rate of 40 frames per second with significantly reduced complexity and setup time, as reported previously [18-20]. The algorithm was designed to superimpose trajectory data onto the first frames of eye movement videos, marking the starting and ending points of the pupil center’s paths. If the algorithm failed to detect the eye due to occlusions or rapid movements, the trajectories were interpolated, and measurement points that could not be captured were omitted from the trajectory path. Additionally, the movement data comprising the x and y coordinates of the pupil at each measurement time point were systematically converted into CSV files for further analysis using LLMs.

This approach proved strategic for our study’s requirements, allowing for rapid development and ease of modification. This was particularly beneficial in our context, in which real-time processing was prioritized over the incremental gains in accuracy afforded by more computationally intensive models.

### GPT-4V Classification With Nystagmus Recording

We developed GPT-4V models to classify eye movement trajectories by generating still images tracked by the algorithm and then inputting these images or CSV data into the GPT-4V model to obtain answers for the classification. When only traced images or coordinates were input, no significant or advantageous responses were obtained. For CSV data inputs containing the pupils’ coordinates (X and Y axes) and their respective measurements, we embedded these data directly into the prompts for the GPT-4 model. We tested three combinations of inputs: only CSV, which used GPT-4 (GPT-4 Turbo); only still images; and a combination of CSV and still images, with the latter two using GPT-4V (GPT-4 Turbo with vision).

A feature of LLMs is in-context learning through prompting. The model was developed using prompts based on previous studies [15,21,22] and the chain-of-thoughts (CoT) prompting technique, which allows LLMs to make complex inferences by entering thought processes into the prompts to facilitate the inference process and reasoning [23,24] (Multimedia Appendix 1). We also used methods, such as metarecognition (MR) and the Rule of 26, which complicates the thought process by making the user aware of the content in reaction to their responses [25,26]. We tested whether the created optimization model could be evaluated in six nystagmus categories: right horizontal, left horizontal, upward, downward, right torsional, and left torsional. Of the trace images obtained from the pupil-tracking process, those with trace points other than the pupil, traces beyond the eyeline, or some areas that could not be traced were excluded by visual inspection by experts. The correctness rate was evaluated for 139 data points (78, 26, and 35 in the horizontal, vertical, and torsional directions, respectively).

An application programming interface was set up for model validation, and the temperature parameter was set to 0 to account for variations in responses. Experts with >20 years of vertigo practice experience judged whether the answers and the explanatory content were appropriate. Even if the final answer was correct, the details that led to the answer were checked, and if they were incorrect, the answer was considered incorrect. Data collected from the video recordings of eye movements exhibiting various nystagmus patterns were used. Each video clip was independently reviewed and annotated by two skilled vertigo specialists to identify the presence and type of nystagmus pattern.

### Ethical Considerations

This study was approved by the Medical Research Ethics Committee of Mejiro University (approval number: Medical 20‐007). Informed consent was obtained from all subjects involved in the study. Written informed consent has been obtained from the patients to publish this paper, as applicable. In accordance with ethical guidelines, an opt-out approach was adopted. Detailed information regarding the study—including its purpose, data handling procedures, and measures for protecting personal information—was made publicly available, and participants were given the opportunity to decline participation if they wished. All data collected in this study were anonymized, ensuring that no personally identifiable information was included in the analysis or publication. No financial or material compensation was provided to the participant.

### Study/Clinical Setting of Recruitment

Participants were recruited in this study from the otolaryngology outpatient clinic at Mejiro University, a tertiary center specializing in the diagnosis and treatment of vestibular disorders. The patient included in this study was referred to the clinic after experiencing episodes of vertigo. During the clinical examination, ocular movements were recorded and the resulting video data were used for this study.

## Results

In this study, we developed a system to classify nystagmus using GPT-4V and obtained the following results.

### Pupil Tracking Process

**Figure 2.**
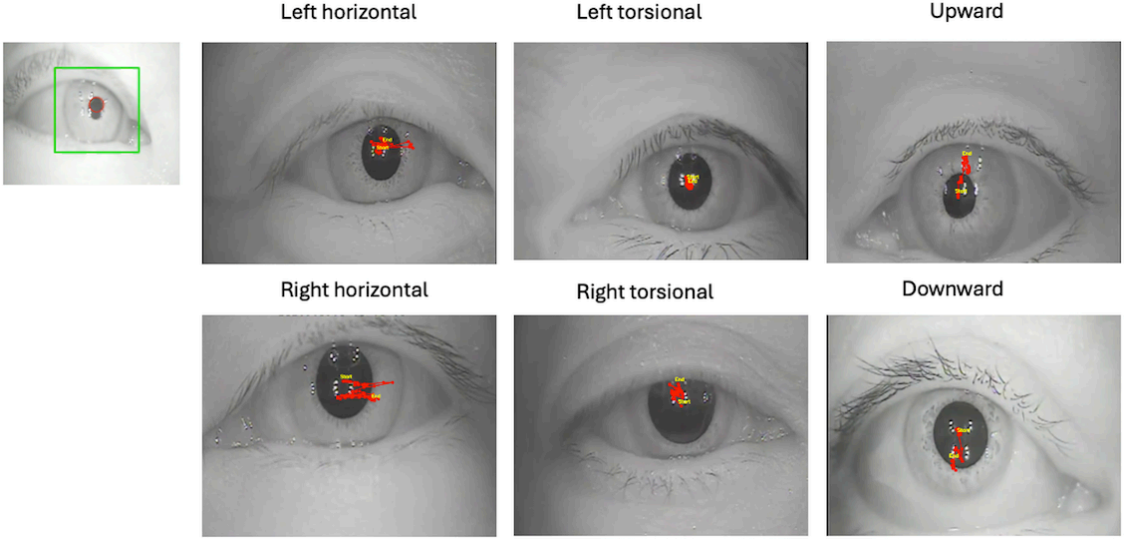
Representative tracing images of six nystagmus types (ie, right horizontal, left horizontal, upward, downward, right torsional, and left torsional) obtained from video-based pupil tracking. Data were collected from patients with vestibular disorders. The images illustrate pupil movement trajectories detected using an AI-based classification model.

The eye movement tracking program accurately recognized the eyeballs in the video and depicted their trajectories (Figure 2). The program successfully tracked eye movements and generated datasets for each nystagmus category.

### GPT-4V Prompting

A nystagmus classification model was constructed by setting and training appropriate prompts on the GPT-4V model. Optimizing the prompts improved the classification accuracy and adapted the model’s response to specific nystagmus patterns. We used both basic and additional prompts for CoT and MR (Multimedia Appendix 1).

### Validation of Nystagmus With GPT-4V

For the “image only” input category, the “basic” prompt method yielded a correct response in 43 (30.9%) instances, whereas no response was recorded in 24 (17.3%) instances (Table 1). The “CSV only” input category under the “basic” prompt approach resulted in 27 (19.4%) correct responses, with 14 (10.1%) instances of no response. When both images and CSV input were used, the “basic” prompt method delivered 37 (26.6%) correct responses, with no response in 12 (8.6%) instances.

**Table 1.** Performance of GPT-4V in classifying nystagmus patterns based on video-recorded eye movements. The table presents the classification accuracy for six nystagmus types across different input modalities (image only, CSV only, and image+CSV). Data were collected from 139 patients diagnosed with vestibular disorders.

Prompting techniques	Input modalities and classification accuracy (N=139)								
	Image only			CSV only			Image+CSV		
	Correct, n (%)	No response, n (%)	Correct response (%)	Correct, n (%)	No response, n (%)	Correct response (%)	Correct, n (%)	No response, n (%)	Correct response (%)
Basic	43 (30.9)	24 (17.3)	37.4	27 (19.4)	14 (10.1)	21.6	37 (26.6)	12 (8.6)	29.1
CoT	47 (33.8)	13 (9.4)	37.3	20 (14.4)	26 (18.7)	17.7	33 (23.7)	32 (23.0)	30.8
MR	50 (36.0)	5 (3.6)	37.3	28 (20.1)	25 (18.0)	24.6	30 (21.6)	33 (23.7)	28.3
CoT+ MR	48 (34.5)	12 (8.6)	37.8	15 (10.8)	69 (49.6)	21.4	35 (25.2)	32 (23.0)	32.7

The other methods showed similar trends, with the CoT prompt approach slightly improving in the “image only” input category with 47 (33.8%) correct responses. Conversely, the MR prompt method outperformed the others in the “image only” input domain, with 50 (36.0%) correct classifications. When combining the CoT and MR prompts, the “image only” input domain showed 48 (34.5%) correct classifications. However, there was a modest improvement in the “image+CSV” input category, with 35 (25.2%) correct responses.

Table 2 shows the outcome of the GPT-4V classification accuracy in the presence of nystagmus-recording data segregated according to each nystagmus direction. Four types of prompting classification strategies were assessed: basic, CoT, MR, and a composite of CoT and MR. The evaluation was further stratified into three data inputs: image only, CSV only, and Image+CSV.

For downward and upward nystagmus, the highest correct classification rates were 37.5% and 27.8%, with a total data count of 8 and 18 instances, respectively.

**Table 2.** GPT-4V classification with nystagmus recording for each direction.

Nystagmus direction	Classification strategies and data input categories											
	Image only (correct classification rates), n (%)				CSV only (correct classification rates), n (%)				Image+CSV (correct classification rates), n (%)			
	Basic	CoT	MR	CoT+MR	Basic	CoT	MR	CoT+MR	Basic	CoT	MR	CoT+MR
Downward (n=8)^a^, n (%)	0 (0)	1 (12.5)	0 (0)	2 (25)	2 (25)	0 (0)	2 (25)	0 (0)	1 (12.5)	0 (0)	0 (0)	3 (37.5)
Left horizontal (n=36)^a^, n (%)	14 (38.9)	14 (38.9)	20 (55.6)	14 (38.9)	3 (8.3)	2 (5.6)	3 (8.3)	0 (0)	9 (25)	7 (19.4)	9 (25)	7 (19.4)
Left torsional (n=16)^a^, n (%)	0 (0)	0 (0)	1 (6.3)	0 (0)	0 (0)	3 (18.8)	1 (6.3)	3 (18.8)	0 (0)	1 (6.3)	0 (0)	0 (0)
Right horizontal (n=42)^a^, n (%)	27 (64.3)	27 (64.3)	25 (59.5)	29 (69.0)	20 (47.6)	15 (35.7)	17 (40.5)	10 (23.8)	23 (54.8)	23 (54.8)	17 (40.5)	22 (52.4)
Right torsional (n=19)^a^, n (%)	1 (5.3)	0 (0)	0 (0)	0 (0)	0 (0)	0 (0)	0 (0)	1 (5.3)	0 (0)	0 (0)	0 (0)	0 (0)
Upward (n=18)^a^, n (%)	1 (5.6)	5 (27.8)	4 (22.2)	3 (16.7)	2 (11.1)	0 (0)	5 (27.8)	1 (5.6)	4 (22.2)	2 (11.1)	4 (22.2)	3 (16.7)

a Total number of nystagmus cases examined.

For left and right torsional nystagmus, the highest correct classification rates were 6.3% and 5.3% with the “image only” inputs, 18.8% and 5.3% with “CSV only” inputs, and 6.3% and 0% with “image+CSV” inputs, respectively.

For the left horizontal nystagmus with 36 data instances, the “image only” input showed superior performance with 38.9% correct classifications using the basic prompt, improving incrementally with the MR prompt at 55.6%. In the right horizontal category with 42 instances, the “image only” input showed a higher correct rate compared to the other input types, with the basic and CoT prompts demonstrating an accuracy rate of 64.3%, while the combined CoT+ MR prompt exhibited the highest accuracy rate at 69%.

The “CSV only” mode indicated a generally lower correct classification rate across all directions and methodologies. Notably, the right torsional and left torsional classifications demonstrated zero correct classifications with basic prompts.

Overall, Table 2 highlights the varying degrees of classification accuracy depending on the direction of nystagmus, data presentation format, and classification method. Inputs containing image data generally showed improved classification performance compared to using CSV only” input.

**Figure 3.**
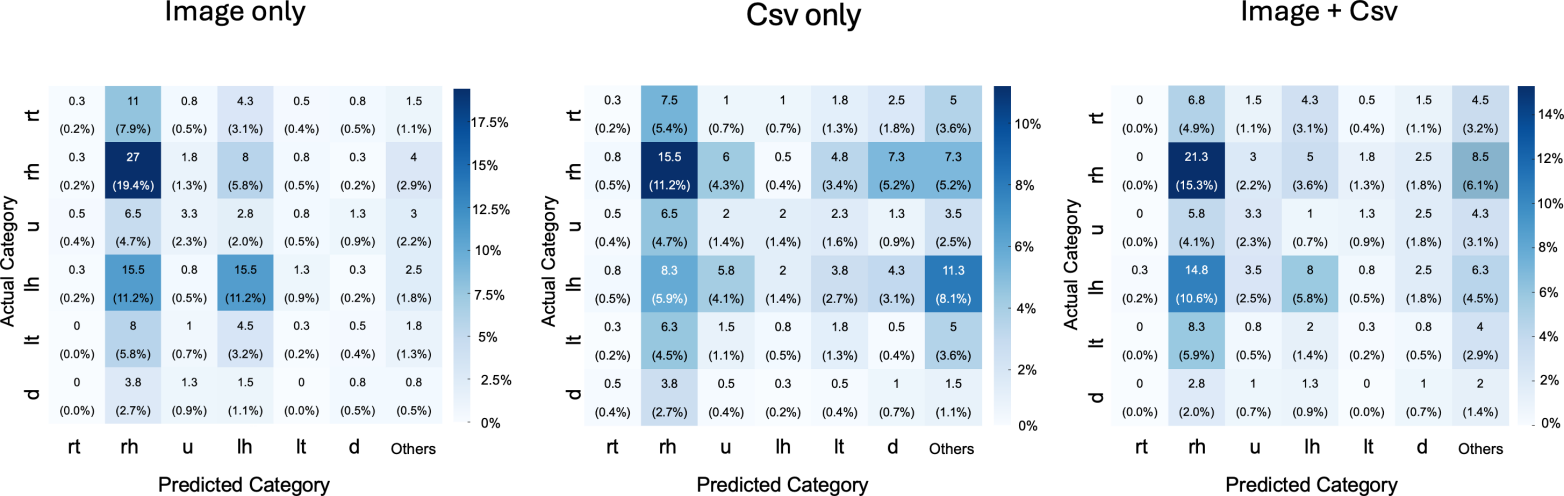
Confusion matrix of GPT-4V classification with nystagmus recording for each direction.

The response trend for each type of data input was analyzed in terms of each direction (Figure 3). The classification performance of the GPT model was evaluated using three different input modalities: image only, CSV only, and image+ CSV. When using the “image only” input, the model achieved an accuracy of 0.289, with a precision of 0.211, a recall of 0.264, and an *F*_1_-score of 0.169. For the “CSV only” input, the accuracy was 0.247, with a precision of 0.218, a recall of 0.210, and an *F*_1_-score of 0.178. The combination of image and CSV inputs (image+ CSV) resulted in the highest performance among the three input types, with an accuracy of 0.356, a precision of 0.186, a recall of 0.222, and an *F*_1_-score of 0.191. The confusion matrix showed a high frequency of horizontal responses, and this tendency was greater for “image only” and “image+ CSV” inputs. For inputs containing images, horizontal nystagmus tended to be misclassified more often, as horizontal nystagmus in the opposite direction occurred more frequently than in the other components. Results for the “CSV Only” input showed a broader distribution of misclassifications across multiple categories, with no specific tendency toward a particular type of nystagmus. Additionally, a notably high number of responses were categorized as “others,” indicating a difficulty in making definitive classifications using CSV data alone. In comparison, the “Image” input demonstrated a reduction in “Others” responses, highlighting improved performance and specificity when combining data modalities.

## Discussion

### Principal Findings

In this study, we created and verified the accuracy of a nystagmus classification model using the GPT-4V. The results revealed several interesting insights. First, it became clear that nystagmus classification is possible using AI generated from a LLM model. Furthermore, the model’s accuracy varied with different prompting adjustments, indicating that the accuracy varied with each nystagmus pattern, suggesting room for improvement in the model’s performance without retraining the model or parameter tuning. Second, it was revealed that inputting images rather than tracking the pupil coordinates resulted in higher accuracy. Thus, the GPT-4V–based nystagmus classification model achieved a certain level of success, and this study serves as the first step toward validating its potential for video analysis.

The GPT-4V-based nystagmus classification model derived from LLM demonstrated the ability to distinguish and classify different nystagmus patterns from video data. Overall, an accuracy rate of 17‐38% was achieved, with the classification of horizontal nystagmus patterns showing a 70% accuracy rate. This indicates that the GPT-4V model can effectively capture subtle differences in horizontal eye movements and suggests specific neurological or vestibular states. The fact that nystagmus classification is possible with an LLM model implies the potential for further development of nystagmus classification models previously tackled using deep learning models. The classification accuracy of deep learning models has been reported to be between 60‐90%, averaging approximately 80%, whereas the GPT-4V similar to LLM, has achieved a certain level of accuracy for horizontal nystagmus [27-29]. As LLMs do not require image training data and can be used in conversations, they can be readily used alongside other information during clinical assessments to confirm answers and diagnostics. At present, LLMs including GPT have improved learning accuracy and can generate still images and audio; however, video recognition has not yet been reported. In the future, our methodology can be applied to videos using the LLM.

Notably, accuracy varies with prompts and conditions [30,31]. We also report that the accuracy of medical licensing and otolaryngology expertise examinations can be improved by presenting choices in English and confirming the examiner’s status [15,18,19]. For the classification of nystagmus, we tested CoT, MR, and their combination, but found no significant improvement in accuracy. GPT is a pretrained general-purpose LLM, and its accuracy is believed to depend on factors such as the number of model parameters, amount of training data, and scale of computational resources [32]. When provided with appropriate prompts, the model may use a greater portion of its parameters more efficiently, generating optimal outputs. Designing appropriate prompts for specific nystagmus patterns is necessary to further increase the model’s discrimination accuracy. Regarding the input methods, the “CSV Only” input showed a notable tendency toward instances where the model frequently failed to classify any type of nystagmus. In contrast, inputs that included image data consistently resulted in producing a specific classification. This indicates that inputs containing images are more effective in enabling the model to provide responses and perform accurate classifications. Moreover, providing multimodal information, such as still images, patient information, and head position is expected to increase the accuracy of balance function tests.

For specific nystagmus patterns, classification was possible for horizontal movements; however, the accuracy of classifying vertical and torsional components was low, regardless of the image or coordinate input. This can be attributed to the evaluation of two dimensional movements, which makes torsional assessment challenging, similar to the limitations of deep learning using two dimensional video capture [27,28]. Additionally, the amplitude of vertical movements was smaller than that of horizontal movements, which could be another reason for lower accuracy. Improving the accuracy and pupil tracking methods with the development of prompts specialized for vertical domains may also be effective for higher precision. Responses showed higher accuracy for identifying horizontal nystagmus compared to other types. These results suggest that the accuracy can be further improved by increasing the input images frame speed, performing more detailed preprocessing, such as changing the settings for each direction, or taking coordinates in three-dimensional directions. This study shows that GPT-4V, a LLM trained on extensive data, can achieve a certain level of accuracy through in-context learning methods such as CoT and MR highlights the broad applicability of this model.

Clinically, nystagmus findings are crucial indicators for evaluating vestibular function and are indispensable in clinical settings for neurological and otologic diseases [5]. However, variability in physician assessments and the occurrence of findings only during vertigo attacks necessitate a stable evaluation model. Electronystagmography, a common method for recording nystagmus clinically, records eye movements as corneoretinal potentials but cannot measure torsional eye movements and has the disadvantage of difficulty in capturing three-dimensional movements. Additionally, the need for specialized equipment makes real-time recording during vertigo attacks challenging, limiting its frequent use in clinical practice. In contrast, methods for recording eye movements using video are becoming widespread [33,34], and with recent advancements in deep learning technology, consistent assessments may become possible. The use of highly versatile LLMs can further expand their application. For example, LLM can be used in written exchanges to confirm repetitions, reasons for thinking so, and corresponding details. The GPT-4V model, with its real-time clinical setting applications, demonstrated faster inference time and lower computational complexity than conventional deep learning techniques. Since some hallucinations may yield incorrect answers as if they were correct, a human must make the decision considering the AI responses [35]. The model’s accuracy, especially in this case, should be limited to cases where a human confirms the answer. Further improvement of the model’s accuracy is required in the future.

As a limitation, this verification was specialized for classifying six types of nystagmus in videos and did not evaluate abnormal detections during regular examinations or nystagmus containing multiple components. Additionally, one limitation is that images being evaluated depend on the nystagmus in videos obtained during the examination, leading to variability in the amount of data depending on the type of nystagmus. Therefore, future studies should include improvements in the classification accuracy of nystagmus patterns and verification of the model’s adaptability to mixed types of clinical data, such as horizontal- and vertical-torsional nystagmus. Moreover, the issue of hallucinations in LLMs is crucial, and how they are used is essential. At a minimum, educational purposes such as training medical professionals and pre-evaluation before doctors can judge the videos, could be effective. Devices capable of recording nystagmus using smartphones exist, and considerations must be made to record and assess patients without medical intervention [36].

The GPT-4V based nystagmus classification model represents significant advancements in medical imaging and diagnostic techniques. Its high accuracy, efficiency, and potential for real-time application make it a valuable tool for improving the diagnosis and management of nystagmus. Continuous research and development in this area are essential for improving the model and maximizing its clinical utility.

### Conclusions

In this study, we developed a nystagmus classification model using GPT-4V and evaluated its performance. Unlike previous deep learning models, GPT-4V, centered on a LLM, presents a promising method for classifying nystagmus in video data and is expected to contribute to improved accuracy and efficiency in medical diagnoses. This represents a significant advance in medical AI and it is crucial to continue refining the model and consider its clinical applications to fully realize the potential benefits that AI technology brings to the medical field.

## Supplementary material

10.2196/70070 Multimedia Appendix 1 Sample of prompt.

## Abbreviations

AI: artificial intelligence
CoT: Chain of Thoughts
GPT-4V: Generative Pre-trained Transformer-4-Vision
LLM: large language model
MR: metarecognition

## References

[1] Ospina LH (2018). Dealing with nystagmus. J Binocul Vis Ocul Motil.

[2] Kates MM, Beal CJ (2021). Nystagmus. JAMA.

[3] Gottlob I (2001). Nystagmus. Curr Opin Ophthalmol.

[4] Newman-Toker DE, Camargo CA, Hsieh YH, Pelletier AJ, Edlow JA (2009). Disconnect between charted vestibular diagnoses and emergency department management decisions: a cross-sectional analysis from a nationally representative sample. Acad Emerg Med.

[5] Bhattacharyya N, Gubbels SP, Schwartz SR (2017). Clinical practice guideline: Benign paroxysmal positional vertigo (update). Otolaryngol--head neck surg.

[6] Lopez-Escamez JA, Gamiz MJ, Fernandez-Perez A, Gomez-Fiñana M (2005). Long-term outcome and health-related quality of life in benign paroxysmal positional vertigo. Eur Arch Otorhinolaryngol.

[7] Santini T, Fuhl W, Kasneci E PuReST: robust pupil tracking for real-time pervasive eye tracking.

[8] Eivazi S, Santini T, Keshavarzi A, Kübler TC, Mazzei A (2019). Improving real-time CNN-based pupil detection through domain-specific data augmentation.

[9] Otero-Millan J, Roberts DC, Lasker A, Zee DS, Kheradmand A (2015). Knowing what the brain is seeing in three dimensions: A novel, noninvasive, sensitive, accurate, and low-noise technique for measuring ocular torsion. J Vis.

[10] Yiu YH, Aboulatta M, Raiser T (2019). DeepVOG: Open-source pupil segmentation and gaze estimation in neuroscience using deep learning. J Neurosci Methods.

[11] Zhang W, Wu H, Liu Y (2021). Deep learning based torsional nystagmus detection for dizziness and vertigo diagnosis. Biomed Signal Process Control.

[12] Kermany DS, Goldbaum M, Cai W (2018). Identifying medical diagnoses and treatable diseases by image-based deep learning. Cell.

[13] Jahan I, Laskar MTR, Peng C, Huang JX (2024). A comprehensive evaluation of large language models on benchmark biomedical text processing tasks. Comput Biol Med.

[14] (2023). GPT-4V(ision) system card. OpenAI.

[15] Noda M, Ueno T, Koshu R (2024). Performance of GPT-4V in answering the Japanese Otolaryngology Board Certification Examination questions: evaluation study. JMIR Med Educ.

[16] Harsha N, Yin TL, Sheng Z, Dean C, Richard E, Nicolo F (2023). Can generalist foundation models outcompete special-purpose tuning? Case study in medicine. arXiv.

[17] Nipun MS, Sulaiman RB, Kareem A (2022). Efficiency comparison of AI classification algorithms for image detection and recognition in real-time. arXiv.

[18] Tian Y, Kanade T, Cohn JF Dual-state parametric eye tracking. https://www.cs.cmu.edu/~face/Papers/fg1camera.pdf.

[19] Padilla R, Costa Filho CF, Costa M (2012). Evaluation of Haar cascade classifiers designed for face detection. World Acad Sci Eng Technol.

[20] Wilson PI, Fernandez J (2006). Facial feature detection using Haar classifiers. J Comput Sci Coll.

[21] Tanaka Y, Nakata T, Aiga K (2024). Performance of generative pretrained transformer on the National Medical Licensing Examination in Japan. PLOS Digit Health.

[22] Noda M, Ueno T, Koshu R (2023). A study of the performance of the generative pretrained transformer in the Japanese Otorhinolaryngology Specialty examination. Nippon Jibiinkoka Tokeibugeka Gakkai Kaiho(Tokyo).

[23] Bsharat SM, Myrzakhan A, Shen Z (2024). Principled instructions are all you need for questioning LLaMA-1/2, GPT-3.5/4. arXiv.

[24] Wei J, Wang X, Schuurmans D, Bosma M, Ichter B, Xia F (2023). Chain-of-thought prompting elicits reasoning in large language models. arXiv.

[25] Touvron H, Lavril T, Izacard G, Martinet X, Lachaux MA, Lacroix T (2023). LLaMA: open and efficient foundation language models. arXiv.

[26] Brown TB, Mann B, Ryder N, Subbiah M, Kaplan J, Dhariwal P (2020). Language models are few-shot learners. arXiv.

[27] Wagle N, Morkos J, Liu J (2022). aEYE: A deep learning system for video nystagmus detection. Front Neurol.

[28] Lim EC, Park JH, Jeon HJ (2019). Developing a diagnostic decision support system for benign paroxysmal positional vertigo using a deep-learning model. J Clin Med.

[29] Lee Y, Lee S, Han J, Seo YJ, Yang S (2023). A nystagmus extraction system using artificial intelligence for video-nystagmography. Sci Rep.

[30] Rao A, Kim J, Kamineni M (2023). Evaluating GPT as an adjunct for radiologic decision making: GPT-4 versus GPT-3.5 in a breast imaging pilot. J Am Coll Radiol.

[31] Chiarelli G, Stephens A, Finati M (2024). Adequacy of prostate cancer prevention and screening recommendations provided by an artificial intelligence-powered large language model. Int Urol Nephrol.

[32] Jared K, Sam M, Tom H, Tom BB, Benjamin C, Rewon C (2020). Scaling laws for neural language models. arXiv.

[33] Bozomitu RG, Păsărică A, Tărniceriu D, Rotariu C (2019). Development of an eye tracking-based human-computer interface for real-time applications. Sensors (Basel).

[34] Cristina S, Camilleri KP (2018). Unobtrusive and pervasive video-based eye-gaze tracking. Image Vis Comput.

[35] Williams CYK, Bains J, Tang T (2024). Evaluating large language models for drafting emergency department discharge summaries. medRxiv.

[36] Fournier-Tombs E, McHardy J (2023). A medical ethics framework for conversational artificial intelligence. J Med Internet Res.

