# Supplementary figures and images for: Exploring Generative Pre-Trained Transformer-4-Vision for Nystagmus Classification: Development and Validation of a Pupil-Tracking Process
Source: JMIR Form Res. 2025 Jun 6;9:e70070. doi: 10.2196/70070 (PMC12164947; doi:10.2196/70070)

Supplementary Material

## Sample of prompt


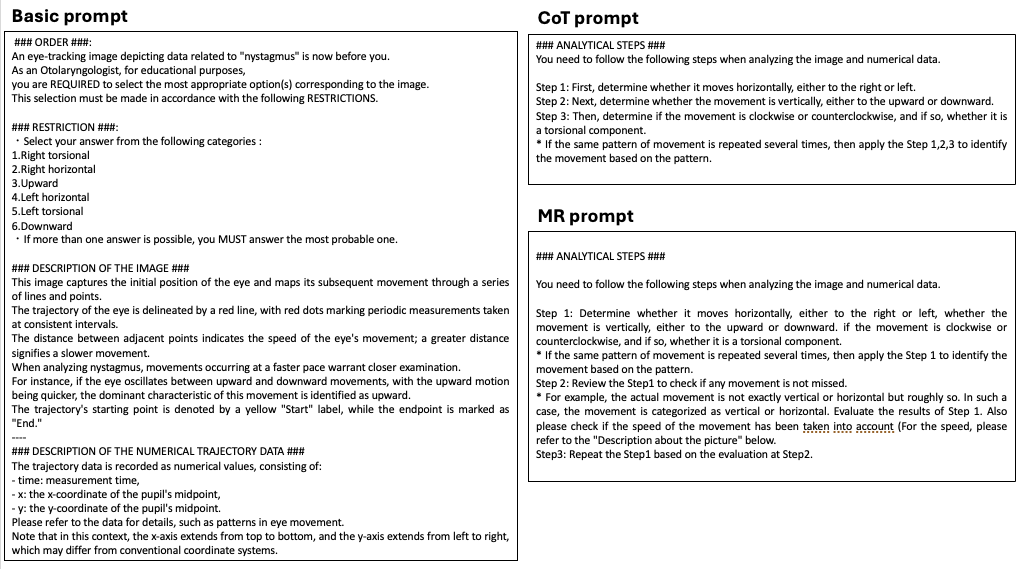

Supplement: Multimedia Appendix 1 [file formative-v9-e70070-s001.docx]
